# The unique calcium chelation property of poly(vinyl phosphonic acid‐co‐acrylic acid) and effects on osteogenesis *in vitro*

**DOI:** 10.1002/jbm.a.36223

**Published:** 2017-09-26

**Authors:** Qi Guang Wang, Ian Wimpenny, Rebecca E. Dey, Xia Zhong, Peter J. Youle, Sandra Downes, David C. Watts, Peter M. Budd, Judith A. Hoyland, Julie E. Gough

**Affiliations:** ^1^ National Engineering Research Center for Biomaterials Sichuan University Chengdu 610064 China; ^2^ Division of Cell Matrix Biology and Regenerative Medicine, Faculty of Biology, Medicine and Health The University of Manchester Manchester M13 9PL United Kingdom; ^3^ School of Materials The University of Manchester Manchester M13 9PL United Kingdom; ^4^ School of Chemistry University of Manchester Manchester M13 9PL United Kingdom; ^5^ Division of Dentistry, School of Medical Sciences and Photon Science Institute University of Manchester Manchester M13 9PL United Kingdom; ^6^ NIHR Manchester Musculoskeletal Biomedical Research Unit, Central Manchester Foundation Trust, Manchester Academic Health Science Centre Manchester United Kingdom

**Keywords:** polyvinyl phosphonic acid, acrylic acid, osteoinductivity, osteoconductivity, calcium chelation

## Abstract

There is a clear clinical need for a bioactive bone graft substitute. Poly(vinyl phosphonic acid‐*co*‐acrylic acid) (PVPA‐*co*‐AA) has been identified as a promising candidate for bone regeneration but there is little evidence to show its direct osteogenic effect on progenitor or mature cells. In this study mature osteoblast‐like cells (SaOS‐2) and human bone marrow‐derived mesenchymal stem cells (hBM‐MSCs) were cultured with PVPA‐*co*‐AA polymers with different VPA:AA ratio and at different concentrations *in vitro*. We are the first to report the direct osteogenic effect of PVPA‐*co*‐AA polymer on bone cells and, more importantly, this effect was dependent on VPA:AA ratio and concentration. Under the optimized conditions, PVPA‐*co*‐AA polymer not only has an osteoconductive effect, enhancing SaOS‐2 cell mineralization, but also has an osteoinductive effect to promote hBM‐MSCs’ osteogenic differentiation. Notably, the same PVPA‐*co*‐AA polymer at different concentrations could lead to differential osteogenic effects on both SaOS‐2 and hBM‐MSCs *in vitro*. This study furthers knowledge of the PVPA‐*co*‐AA polymer in osteogenic studies, which is critical when utilizing the PVPA‐*co*‐AA polymer for the design of novel bioactive polymeric tissue engineering scaffolds for future clinical applications. © 2017 The Authors Journal of Biomedical Materials Research Part A Published by Wiley Periodicals, Inc. J Biomed Mater Res Part A: 106A: 168–179, 2018.

## INTRODUCTION

Bone is a type of tissue with a self‐healing capability. However, bone fractures beyond the critical size: 3 cm for the forearm, 5 cm in the femur and tibia, 6 cm in the humerus; are always associated with impaired healing, resulting in a delayed union or nonunion.^1^ With large bone defects, post‐traumatic injuries or revision total hip replacement surgeries, surgery requires bone grafts/void fillers to be placed between bones or the bone and prosthesis, to bridge the gap or stabilize and promote bone growth and integration. Consequently, bone grafting is a common surgical procedure, and it has been estimated >2 million grafting procedures are performed worldwide each year.^2^ Hence there is a clear clinical need for bioactive bone void filler.

Tissue engineering is a promising strategy to create bioactive bone void filler. It uses biocompatible and biodegradable materials to provide temporary structural support and to stimulate new bone growth. With the recent technology advancements on scaffold biological functionality, researchers have focused on creating a bioactive scaffold that mimics the native bone extracellular matrix with both osteoconductive and osteoinductive properties.^3^ This can be achieved through the incorporation of bioactive moieties, such as polymers, growth factors, or proteins, into or onto the scaffold.^4^, ^5^, ^6^, ^7^, ^8^

Poly vinyl phosphonic acid‐*co*‐acrylic acid (PVPA‐*co*‐AA) has been identified as a potential candidate to enhance bone growth by incorporating it within a scaffold. It has been hypothesized to mimic the action of bisphosphonates, due to a similar chemical structure and the ability of the phosphonate group to chelate Ca^2+^ ions, essentially forming a “bone hook,” with the potential to enhance bone mineralization.^9^ Previous studies utilized PVPA‐*co*‐AA as a surface treatment of electrospun tissue engineering scaffolds, demonstrating improved surface wettability, compressive strength, cell viability and bone formation.^10^, ^11^, ^12^ However, factors such as scaffold topography, porosity, pore size, interconnectivity, and so forth can also contribute to the osteogenic effect of a scaffold,^13^ and thus it is unclear whether the effect in these previous studies was a result of a direct osteogenic effect on cells, or improved wettability of the scaffold surface caused by the addition of the PVPA‐*co*‐AA polymer. Furthermore, conflicting results have also been reported in that the PVPA‐*co*‐AA treated samples induced little to no mineralization on collagen scaffolds.^14^ Hence, the exact nature by which PVPA‐*co*‐AA influences bone regeneration needs to be further investigated to realize its clinical potential and the mode of action of PVPA‐*co*‐AA following incorporation into bioactive bone grafts.

**Figure 1:**
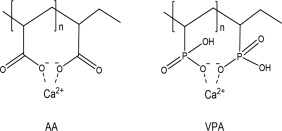
Calcium chelation ability of AA and VPA. In PAA, Calcium binds to the carboxylate groups from neighboring polymer segments. In PVPA, VPA performs as a monoprotic acid and it is the close proximity of the phosphonate groups, coupled with the strong negative charge of ions, which chelate the calcium ions.

PVPA‐*co*‐AA is derived from two monomers, vinyl phosphonic acid (VPA) and acrylic acid (AA), and thus has both phosphonic acid and carboxylic acid functional groups. Both of these are capable of chelating calcium (Fig. 1) and have been previously used in bone tissue engineering scaffolds. Poly vinyl phosphonic acid (PVPA) has been utilized with chitosan or polyvinyl alcohol scaffolds to treat rat calvarial defects, and more pronounced tissue growth and bone formation was observed after *in vivo* implantation.^10^, ^15^ Poly acrylic acid (PAA) has been used within chitosan–silica hydrogels carrying platelet gels for bone defect repair^16^ and nanocomposites of PAA nanogels and biodegradable polyhydroxybutyrate have been employed in bone regeneration and drug delivery.^17^ Therefore, both the VPA and AA units in the PVPA‐*co*‐AA polymer may enhance bone regeneration, and the optimized combination/ratio in the PVPA‐*co*‐AA copolymer requires further understanding.

The objective of this study was to determine if there is a direct biological effect of PVPA‐*co*‐AA on bone cells, including cell viability, metabolic activity, alkaline phosphatase activity, collagen synthesis and the production of a mineralized matrix *in vitro*. As both the VPA and AA components in the copolymer have an ability to chelate calcium, hence promoting bone mineralization, we hypothesize that different VPA:AA ratios in the PVPA‐*co*‐AA polymer may lead to differential osteogenic effects. In particular, we investigated the osteoinductive and osteoconductive effect of PVPA‐*co*‐AA polymers using the human bone marrow derived mesenchymal stem cells (hBM‐MSCs) and an osteoblast cell line, SaOS‐2 cells.

## MATERIALS AND METHODS

### Materials

PVPA‐*co*‐AA polymers with different AA and VPA ratios were synthesized in house.^18^ Six PVPA‐*co*‐AA polymers with different AA‐VPA ratios (mol %) of 0:100 (PAA), 22:78 (P‐22), 34:66 (P‐34), 56: 44 (P‐56), 78:22 (P‐78), and 100:0 (PVPA) (Table 1) were used in this study. Prior to cell culture, all PVPA polymers were diluted to 10 mg/mL concentration in PBS and adjusted to pH 7.3, followed by autoclave sterilization.

**Table 1:** Six PVPA‐*co*‐AA Polymers with Different AA‐VPA Ratios (mol %)

Sample Code	Monomer Feed Ratio (VPA:AA)	Copolymer Composition (VPA:AA)	Molecular Weight (g mol^−1^)
PAA	0:100	0:100	362,000
P‐22	30:70	22:78	267,000
P‐34	40:60	34:66	18,200
P‐56	60:40	56:44	19,700
P‐78	80:20	78:22	9400
PVPA	100:0	100:0	9400

### Cell culture

Human bone marrow was removed from the proximal femur during hip‐replacement surgery after approval from the North West Research Ethics Committee and with fully informed written consent of patients (male, *n* = 3; average age, 52 years (age range, 48 to 54 years)). hBM‐MSCs were isolated and expanded in Minimum Essential Media α (α‐MEM) (Sigma‐Aldrich, UK) with 20% FCS, as previously described.^19^ After 5 days, nonadherent cells were discarded, and adherent cells were cultured to confluence. hBM‐MSCs were subsequently cultured in α‐MEM supplemented with 10% FCS, antibiotics (100 U/mL Penicillin, 100 mg/mL streptomycin), 1% Glutamax and 10 uM ascorbic acid (referred to as hBM‐MSCs CCM). hBM‐MSCs at passage 3 were used for subsequent experiments.

Human osteosarcoma derived osteoblast cells (SaOS‐2) were purchased from Sigma (Sigma‐Aldrich, UK) and cultured in T75 flasks with Dubecco's modified Eagles media (DMEM) (Sigma‐Aldrich, UK) supplemented with 10% fetal calf serum (FCS), antibiotics (100 U/mL Penicillin, 100 mg/mL streptomycin) and 50 uM ascorbic acid (referred to as SaOS‐2 CCM). They were maintained at 37°C in a humidified atmosphere of 95% air and 5% CO_2_.

### Cell viability and metabolic activity

SaOS‐2 cells were seeded into 24‐well plates, at a density of 40,000 cells per well. After 24 hours a range of PVPA‐*co*‐AA concentrations (0 to 1 mg/mL) were then added to human SaOS‐2 cells cultures for 7 days. The media was replenished every 2–3 days, and cell viability and metabolic activity was measured at day 0, 1, 3, and 7.

Cell viability was assessed using the Live/Dead Cell Double Staining Kit (Sigma‐Aldrich, Poole). The kit contains calcein‐AM and propidium iodide, which stain viable and nonviable cells, respectively.^20^ Fluorescence was detected at 490 nm excitation for simultaneous monitoring of viable and dead cells. Two hundred cells were counted in three separate regions of each well (4 replicates). Percentage viability was assessed by dividing the number of viable cells by the total number of counted cells.

For cell metabolic activity, the AlamarBlue® Cell Viability Reagent (Invitrogen, UK) was used.^21^ In brief, initial metabolic activity (day 0) was measured using the Alamar Blue assay, before 0–500 μg/mL of the PVPA‐*co*‐AA solution was added to the culture media. Phosphate buffered saline (PBS) was used as a control and a number of PVPA‐*co*‐AA solutions were tested, with VPA contents ranging from 0 to 100 mol % at the different concentrations from 10 to 1000 µg/mL. Cell metabolic activity was then measured at 24 h and 72 h time points. The resulting fluorescence was read on a plate reader (FLx800, Biotek, UK) with an excitation wavelength of 540 nm and an emission wavelength of 600 nm. The fold change in SaOS‐2 cell number was calculated by comparing the cell number at day 1, day 3 and day 7, to day 0 (3 replicates). The statistical difference between each treatment and the PBS control was compared using the one‐way ANOVA analysis.

### Calcium chelation test

The amount of Ca^2+^ chelated by the PVPA‐*co*‐AA polymer was measured using a calcium‐ion‐selective electrode (calcium ISE) from ThermoFisher Scientific, UK. A range of PVPA‐*co*‐AA copolymers (5 mg mL^−1^) were dissolved in 0.1 M NaCl solutions in deionized water and neutralized to the required pH 7.3 using 0.1 M NaOH. CaCl_2_ (0.1 M) was added to the polymer solutions which were then stirred for 30 min The calcium ISE was then immersed into the polymer solutions to measure the free Ca^2+^ concentration. A calibration curve was produced prior to the measurement using a range of CaCl_2_ standard solutions (0.0001, 0.001, 0.01, 0.1, and 1 M). The samples were then quantified by means of the calibration curve (*R* ^2^ > 0.999). The amount of polymer‐bound calcium was calculated from the total amount of Ca^2+^ added and the amount of free Ca^2+^ measured.

### PVPA‐*co*‐AA treatment in osteogenic media

hBM‐MSCs were seeded in 48‐well plates at a density of 20,000 cells per well in 0.5 mL hBM‐MSCs CCM until they reached confluence (usually after 72 h). To promote osteogenic differentiation, the media was then replaced with the hBM‐MSCs osteogenic media (hBM‐MSCs OM), which was α‐MEM supplemented with 10% FCS, antibiotics (100 U/mL Penicillin, 100 mg/mL streptomycin), 1% Glutamax, 10 nM dexamethasone, 50 μg/mL L‐ascorbic acid and 5 mM inorganic phosphate (Sigma‐Aldrich, UK).

SaOS‐2 cells were seeded in 24‐well plates at a density of 40,000 cells per well in 1 mL SaOS‐2 CCM until they reached confluence. After 72 h media was replaced with the SaOS‐2 osteogenic media (SaOS‐2 OM), which was the DMEM supplemented with 0.5% FCS, antibiotics (100 U/mL Penicillin, 100 mg/mL streptomycin), 10 nM dexamethasone and 50 μg/mL L‐ascorbic acid (Sigma‐Aldrich, UK). Two days prior to the end of the experiment, 5 mM inorganic phosphate was added to the osteogenic media to promote mineralization.^22^

PVPA‐*co*‐AA polymers with various AA‐VPA ratios at a range of concentrations (0 to 100 µg/mL) were added into the corresponding osteogenic cultures throughout the entire period, and replenished every 2–3 days with the media change. hBM‐MSCs were cultured in hBM‐MSCs OM up to 28 days and SaOS‐2 cells were cultured in SaOS‐2 OM up to 7 days, while various analyses were performed at different time points.

### Alkaline phosphatase assay

hBM‐MSCs samples in hBM‐MSCs OM were analyzed at day 14 and SaOS‐2 cell samples in SaOS‐2 OM were analyzed at day 7. The cells were washed with PBS and fixed in 4% paraformaldehyde for 1 min. Cells were then washed once with PBS containing 0.05% Tween and stained with BCIP/NBT assay solution (SigmaFast^TM^ BCIP‐NBT; Sigma Aldrich) for 10 min. The plates were washed three times with PBS with 0.05% Tween and air‐dried prior to scanning on a flatbed scanner. The percentage area of mineralization per well was quantified using Image J (http://imagej.nih.gov/ij/) and expressed as percentage response to different PVPA‐*co*‐AA treatments.

### *In vitro* mineralization assay

hBM‐MSCs samples in hBM‐MSCs OM were analyzed at day 21 and SaOS‐2 cell samples in SaOS‐2 OM were analyzed at day 7. The cells were fixed overnight in 4% paraformaldehyde and stained with 40 mM Alizarin Red S (pH 4.2, Sigma‐Aldrich, UK) for 45 min. The plates were washed four times with distilled water and air‐dried prior to scanning on a flatbed scanner. The percentage area of mineralization per well was quantified using Image J and expressed as percentage response to different PVPA‐*co*‐AA treatments.

### Picrosirius red collagen assay

hBM‐MSCs samples in hBM‐MSCs OM were analyzed at day 28 and SaOS‐2 cell samples in SaOS‐2 OM were analyzed at day 7. The cells were fixed in 100% ethanol overnight at −20°C. The cells were washed once with PBS and incubated in PicroSirius Red staining solution (0.1%) (Sigma‐Aldrich, UK) at room temperature for 1 hour. The staining solution was removed, and the samples were washed three times with 0.1% acetic acid to remove the excessive staining residues.^23^ The plates were washed by PBS with 0.05% Tween three times and air‐dried prior to scanning on a flatbed scanner. The percentage area of mineralization was quantified using Image J and expressed as percentage response to different PVPA‐*co*‐AA treatments.

### Scanning electron microscopy (SEM)

The structures of calcium‐chelating polymers and the morphology of the cells cultured on Thermanox® coverslips and polymer samples were observed by scanning electron microscopy. Calcium chelating polymers were prepared in a CaCl_2_ solution at concentrations in excess of the maxiumum chelation capacity of each polymer (PAA, P‐34 and PVPA). Calcium‐polymer complexes were centrifuged (5 min at 5000 rpm). The pellet was re‐suspended in 100% ethanol and centrifuged and the process repeated twice with a final wash in dH_2_O. Samples were frozen at −80°C and freeze dried for 24 h. Samples were adhered to aluminum stubs with conductive carbon tape and sputter‐coated with gold (Edwards, UK) prior to imaging. Calcium‐chelated polymers were observed using a Phenom G2 Pro desktop scanning electron microscope (SEM; Eindhoven, Netherlands) with an accelerating voltage of 5 kV. Samples that had been cultured with cells were imaged using a variable pressure SEM (Zeiss EVO60; Carl Zeiss Ltd, UK).

### Quantitative real‐time PCR

hBM‐MSCs samples in hBM‐MSCs OM were analyzed at days 0, 21 and 28, while SaOS‐2 cell samples in SaOS‐2 OM were analyzed at days 0, 1 and 5. At each time point, the cells were washed by PBS twice and lysed with 1 mL TRIzol reagent (Life Technologies, UK), then incubated for 5 min with constant agitation. After incubation, the RNA was extracted as previously described,^24^ quantified using a Nanodrop and reverse‐transcribed to cDNA, using a High Capacity cDNA Reverse Transcription Kit (Life Technologies, UK). Real‐time quantitative polymerase chain reaction (PCR) was performed on a StepOnePlus real‐time PCR system (Life Technologies, UK), using Lumino‐Ct qPCR ReadyMix (Sigma‐Aldrich, UK).

Assays were prepared using FAM‐BHQ1 assays (all primers and probes from Sigma‐Aldrich) for the following genes: alkaline phosphatase (ALPL; NM_000478), forward ACGTCTTCACATTTGGTG, reverse GGTAGTTGTTGTGAGCATA; collagen type I (COL1; NM_000089), forward TCAGCTTTGTGGATACGC, reverse CTGGGCCTTTCTTACAG; osteopontin (OP; NM_000582), forward CTGACATCCAGTACCCTG, reverse CAGCTGACTCGTTTCATA; runt‐related transcription factor 2 (RUNX2; NM_001024630), forward CGCTGCAACAAGACC, reverse CGCCATGACAGTAACC; osteocalcin (OC; NM_199173), forward CCGCACTTTGCATCG, reverse GCC‐ATTGATACAGGTAGC. The gene expressions were normalized to housekeeping gene glyceraldehyde 3‐phosphate dehydrogenase (GAPDH; NM_001256799), Forward CTCCTCTGACTTCAACAG, Reverse CGTTGTCATACCAGGAA; and represent mean ± SD.

## RESULTS

### Optimized PVPA‐*co*‐AA concentrations for cell culture

**Figure 2:**
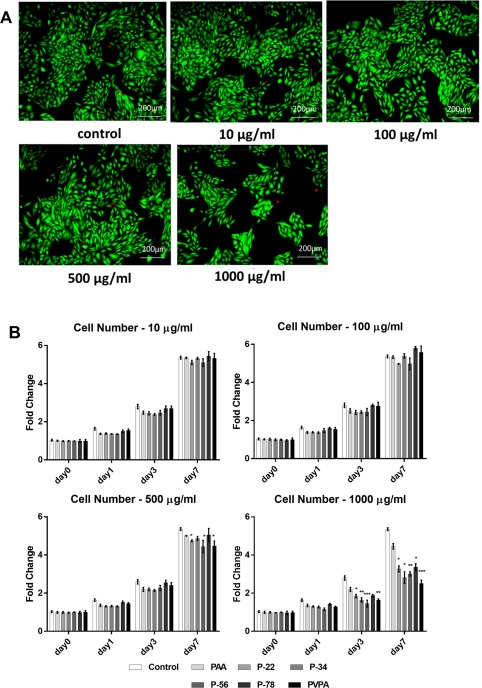
SaOS‐2 cell viability and cell number subject to different PVPA‐co‐AA polymer treatments at different concentrations (A) Representative SaOS‐2 cells subject to a range of PVPA‐co‐AA polymer treatments at different concentrations stained with fluorescent Live/Dead Double Staining at day 7 day; live cells appear green, dead cells appear red. (B) SaOS‐2 cell number subject to PVPA‐co‐AA polymer treatments at different concentrations measured by AlamarBlue® Cell Viability assay at day 0, 1, 3, 7. The graph shows means ± SD of data. Asterisks indicate significant (*p < 0.05, **p < 0.01, ***p < 0.001) difference in fold change vs. the control at the same time point.

Viable SaOS‐2 cells were observed at all test PVPA‐*co*‐AA concentrations after 7 days’ culture, with the cell viability above 95% in all test samples. However, there were visually fewer cells with the 1000 µg/mL PVPA‐c*o*‐AA treatment [Fig. 2(A)]. Figure 2(B) shows that compared to PBS control, the cell metabolic activity was not significantly different at PVPA‐*co*‐AA polymer concentrations of 10 µg/mL and 100 µg/mL throughout the 7‐day culture period. In contrast, at 500 µg/mL concentration, the cell metabolic activity was significantly decreased compare to control with the P‐22, P‐56, and PVPA treatment at day 7. At 1000 µg/mL concentration, the cell metabolic activity was significantly decreased with the P‐22, P‐34, P‐56, and PVPA treatment at day 3 and day 7; and P‐78 treatment at day 7. In contrast, no significant difference in cell metabolic activity was found with the PAA treatment compare to PBS control. Overall, all PVPA‐*co*‐AA treatments at concentrations of 100 µg/mL or lower showed no effect on cell metabolic activity up to 7 days. Hence PVPA‐*co*‐AA concentrations were kept below 100 µg/mL in subsequent cell work.

### The unique calcium chelation property of the PVPA‐*co*‐AA polymer

**Figure 3:**
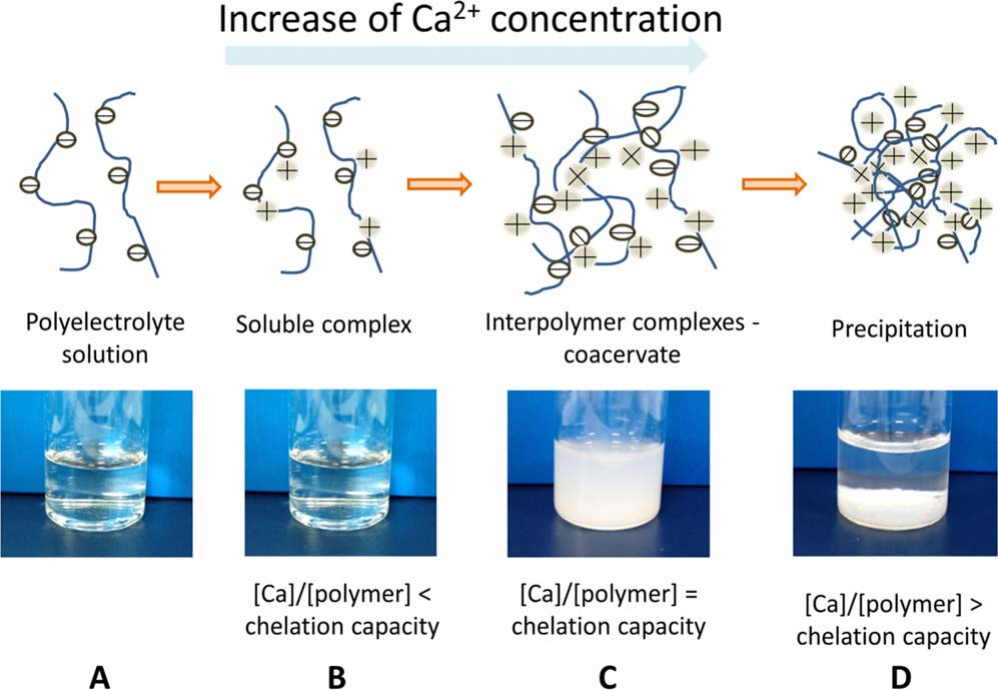
Calcium chelation mechanism of the PVPA‐co‐AA polymer (A) PVPA‐co‐AA copolymer dissolves in aqueous solution to form a negatively‐charged polyelectrolyte. (B) PVPA‐co‐AA can bind to divalent calcium ions. (C) At high calcium concentrations, inter‐polymer complexes can be formed, resulting in a cloudy solution. (D) Precipitation of polymer‐calcium complex at very high calcium concentrations.

PVPA‐*co*‐AA is a polyelectrolyte of intermediate strength. Figure 3(A) illustrates the dissociation of phosphonic and carboxylic acid groups to give a negatively‐charged polyelectrolyte in aqueous solution. PVPA‐*co*‐AA can chelate calcium ions to form a clear, soluble complex. This occurs when the capacity of the polymer is higher than the calcium concentration added into solution [Fig. 3(B)]. When the capacity of PVPA‐*co*‐AA is equal to the calcium concentration, interpolymer complexes are formed, producing a cloudy solution [Fig. 3(C)]. Finally, an overabundance of calcium ions in solution will lead to precipitation of the polymer‐calcium complex [Fig. 3(D)].

**Figure 4:**
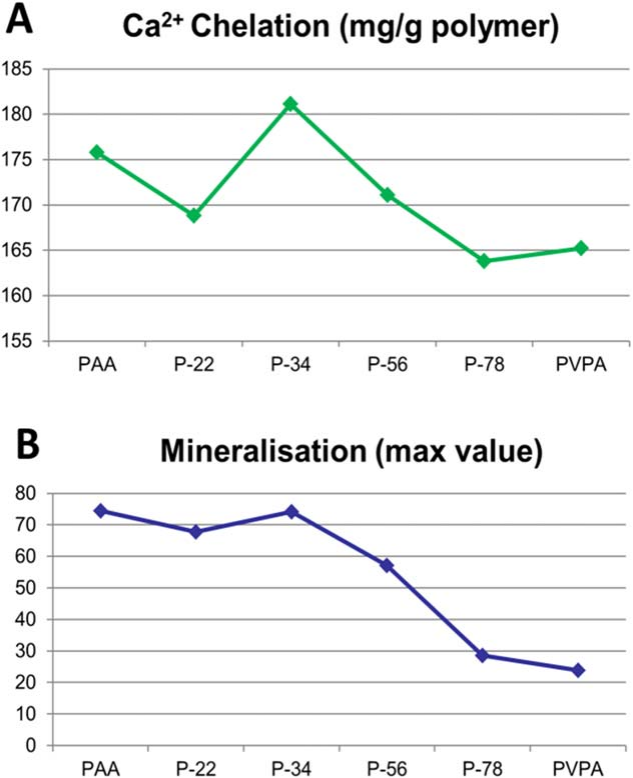
(A) Calcium chelation capacity of the different PVPA‐co‐AA polymer at pH 7.3 and (B) the maximum value of SaOS‐2 cells mineralization with the different PVPA‐co‐AA polymer treatments.

The calcium chelation capacity of each PVPA‐*co*‐AA polymer was assessed [Fig. 4(A)] Calcium chelation increased with VPA content from 22% to 34%, after which there was a decrease with increasing VPA mol %.

### The VPA:AA ratio of PVPA‐*co*‐AA polymer affected the amount of mineralization in SaOS‐2 cells

**Figure 5:**
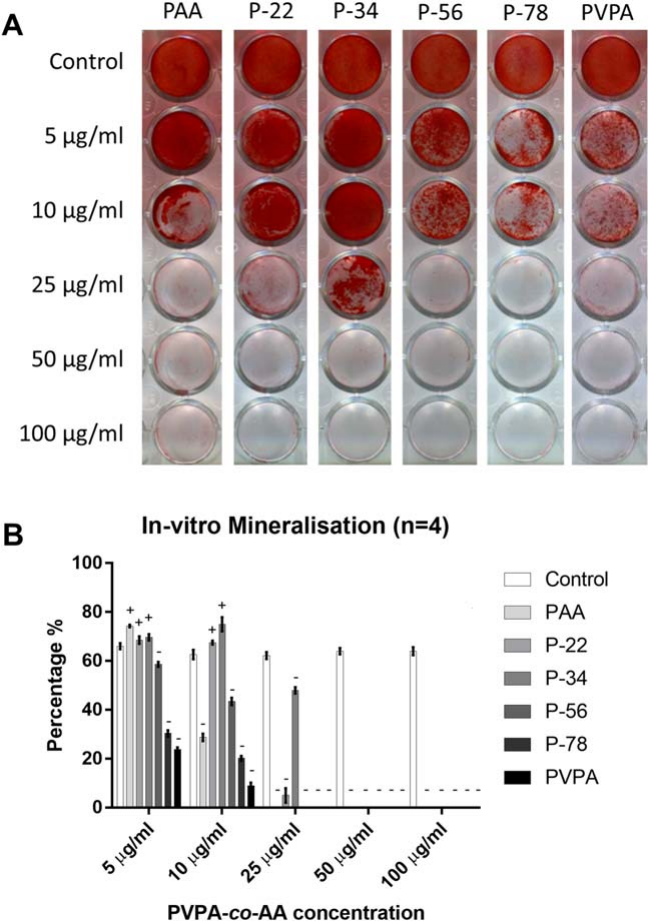
Effect of different PVPA‐co‐AA polymer treatments at different concentrations on SaOS‐2 cells mineralization. (A) Representative photos showing the patterns of alizarin red S staining of the *in vitro* mineralization matrix subject to PVPA‐co‐AA polymer treatments at different concentrations. (B) Shows the quantified percentage of SaOS‐2 cells mineralization subject to PVPA‐co‐AA polymer treatments at different concentrations. The graph shows means ± SD of data. ± indicates the significant increase or decrease (at least *p* < 0.05) in the mineralization percentage vs. the control samples.

The 7‐day *in vitro* mineralization staining patterns with various PVPA‐*co*‐AA treatments are shown in Figure 5(A) with quantitation depicted in Figure 5(B). With 5 µg/mL PVPA‐*co*‐AA treatment, three polymers; PAA, P‐22, and P34 showed significantly higher mineralization than the control while the other polymers showed significantly lower mineralization than the control. With 10 µg/mL PVPA‐*co*‐AA treatment, two polymers; P‐22, and P34 showed significantly higher mineralization than the control while the other polymers showed significantly lower mineralization than the control. With PVPA‐*co*‐AA treatment at 25 µg/mL or above, all treatment samples showed significantly lower or even no mineralization at all, compared to the control. Similar to the calcium chelation capacity, the cell mineralization was increased with VPA content from 22% to 34%, after which there was a decrease with increasing VPA% [Fig. 4(B)].

### The VPA:AA ratio of PVPA‐*co*‐AA polymer affected the cell mineralization pattern

**Figure 6:**
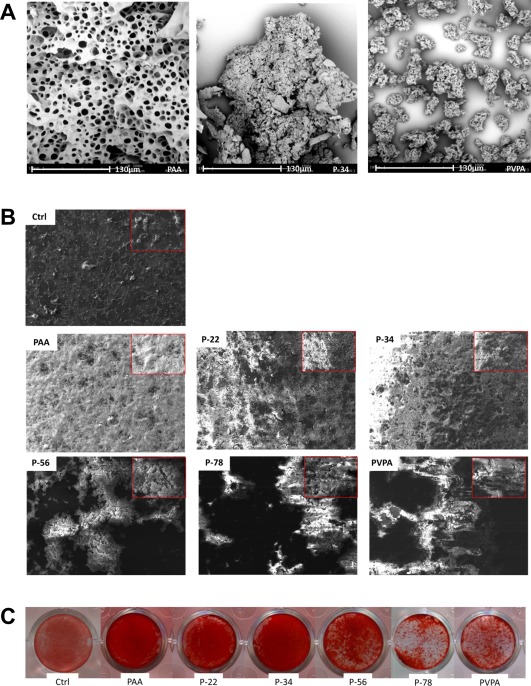
Representative SEM images (A and B) and a comparison with Alizarin red mineralization staining (C). SEM images demonstrate the differences in the polymer‐calcium complexes, indicative of the chelation capacities of the different polymer ratios, whereby the increasing amount of VPA (0, 34, and 100 mol % VPA) show increased levels of granulation/particle formation when exposed to the same concentrations of Ca2+ ions (A). The subsequent effects of the different AA:VPA ratio on the culture of SaOS‐2 cells, is demonstrated with a lower magnification image is inset (B); the differences in particle formation of morphology of particles was considered to play a role in the mineralization behavior in association with cells. Dark areas are indicative of acellular regions on the surface of the thermanox coverslip and appear featureless in contrast with the bright areas of mineralized material. In comparison to the SEM the calcium deposition observed by SEM could be correlated to the mineralization observed by alizarin red staining for calcium (C), where increasing the mol % VPA resulted in more agglomerated clusters of mineralization, whereas P‐34 demonstrated a much more homogenous layer of mineralization with the presence of nodulelike formations.

The solutions themselves had very different appearances when observed visually. For example, PAA produced a rather turbid solution, P‐34 was less cloudy with some precipitate and PVPA demonstrated a clear supernatant with a stable precipitate at the base of the centrifuge tube. This was thought to indicate that interpolymer‐calcium complexes may possess different properties, in terms of propagation of particle formation and potential for ionic exchange. The calcium‐interpolymer complexes of PAA, P‐34, and PVPA‐*co*‐AA polymers were freeze dried and investigated by SEM to observe the difference in the physical morphology. Distinct differences in the complexes could be observed by SEM (Fig. 6). PAA appeared to be a flaky, porous polymer film. It was considered that the pores were created as a result of sublimation of the ice crystals during the process of freeze drying. However, P‐34 appeared to have formed distinct large particles. It appeared that smaller particles may have acted as nucleation sites and the particles continued to agglomerate to form relatively large, dense clusters, with an appearance similar to bone nodules [Fig. 6(A)]. Finally, the PVPA homo‐polymer also demonstrated the formation of particulates, but they appeared smaller and better dispersed (limited agglomeration). The size and shape of calcium phosphate particles and their chemistry is known to affect the cell‐bioceramic interaction, causing changes in protein expression and affecting further mineralization of the surface.

The chelation capacity of the pure constituent polymers and the ratio of these constituents in the copolymers clearly affected the morphology of the mineralized component that can interact with the cells. The extent and morphology of the mineralized matrix was observed in association with SaOS‐2 cells after 7 days’ culture in osteogenic media. All the polymer‐associated samples demonstrated regions of mineralized matrix production. However, relative to the control (thermanox coverslip), the matrix was much more densely packed [Fig. 6(B)]. PAA, P‐22, and P‐34 presented similar levels of confluence and matrix distribution across the surface of the coverslips. However, as the molar ratio of VPA increased from 34 mol %, the deposition of matrix was less homogenously distributed, with a tendency to form clusters of mineralized matrix, leaving large areas of the underlying coverslip exposed. This may be as a result of cells adhering to the larger, distinct polymer‐calcium complexes, previously observed in the polymer‐calcium complexes in solution. These observations were consistent with the distribution of positive (red) alizarin red staining for the presence of calcium [Fig. 6(C)].

Since P‐34 showed the best overall results in calcium chelation capacity and amount of mineralization in culture experiments, indicating the optimized VPA:AA ratio among all tested polymers, it was selected for further study.

### Osteogenic effect of P‐34 on hBM‐MSCs was concentration dependent

**Figure 7:**
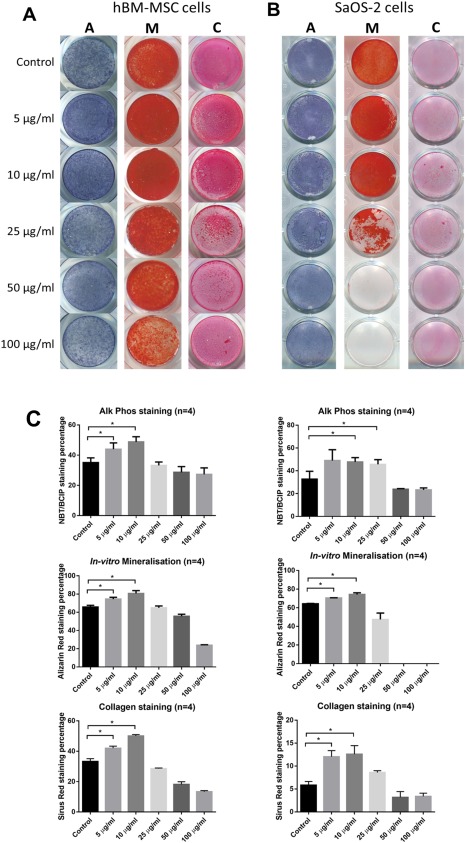
Osteogenic effect of PVPA‐co‐AA polymer in SaOS‐2 cells and human BM‐MSCs. Representative photos showing the patterns and quantified percentage of ALP, in‐vitro mineralization, and collagen staining of (A) human BM‐MSCs and (B) SaOS‐2 cells subject to P‐34 polymer treatments at different concentrations. The graph shows means ± SD of data. Asterisks indicate significant (*p < 0.05, **p < 0.01, ***p < 0.001) increase in the staining percentage vs. the control at the same time point.

hBM‐MSCs cells were cultured in osteogenic media with different concentrations of PVPA (P‐34) for 28 days and alkaline phosphatase activity at day 14, *in vitro* mineralization and collagen synthesis at day 28 were assessed [Fig. 7(A)]. The results show that at 5 µg/mL and 10 µg/mL concentrations, P‐34 treatment significantly increased alkaline phosphatase activity, *in vitro* mineralization, and collagen synthesis at the relevant time points.

### Osteogenic effect of P‐34 on SaOS‐2 cells was concentration dependent

SaOS‐2 cells were cultured in osteogenic media with different concentrations of PVPA (P‐34) for 14 days and alkaline phosphatase activity and *in vitro* mineralization assessed at day 7; and collagen synthesis assessed at day 14 [Fig. 7(B)]. The results show that at 10 µg/mL and 25 µg/mL concentrations, P‐34 treatment significantly increased alkaline phosphatase activity at day 7. At 5 µg/mL and 10 µg/mL concentrations, P‐34 treatment significantly increased *in vitro* mineralization at day 7 and the collagen synthesis at day 14.

### P‐34 significantly increased osteogenic gene expression in hBM‐MSCs

**Figure 8:**
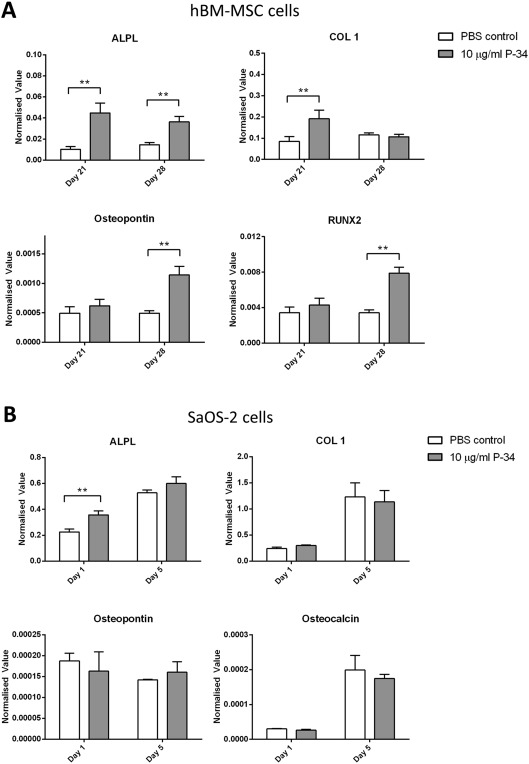
Osteogenic marker gene expression in SaOS‐2 cells and human BM‐MSCs. (A) shows the osteogenic marker gene expression in human BM‐MSCs at day 21 and 28, subject to P‐34 polymer treatments at different concentrations. (B) shows the osteogenic marker gene expression in SaOS‐2 cells at day 1 and 7, subject to P‐34 polymer treatments at different concentrations. The data were normalized to housekeeping gene GAPDH rRNA and represent mean ± SD. Asterisks indicate significant (*p < 0.05, **p < 0.01, ***p < 0.001) difference between the samples at the same time point.

Human hBM‐MSCs treated with P‐34 showed increased expression of all genes compared to the PBS control [Fig. 8(A)]. The osteogenic marker gene ALPL was significantly higher in the treatment group at both day 21 and day 28; COL1 was also significantly increased at day 21 in the P‐34 treated samples. RUNX2 and OP both showed a significant increase at day 28 in samples treated with P‐34. The mature osteoblast marker gene OC was not detected in any day 21 samples and only detected in less than half of the day 28 samples after 35/40 PCR cycles and thus results were not analyzed.

P‐34 increased ALPL gene expression in SaOS‐2 cells at day 1 but no other gene expression was significantly increased compared to PBS control. Osteogenic gene expression in SaOS‐2 cells was not increased with P‐34 treatment [Fig. 8(B)] throughout the 7‐day culture period.

## DISCUSSION

In this study, a range of PVPA‐*co*‐AA polymers with the VPA:AA ratio from 0 to 100 have been tested; no cytotoxicity was seen at concentrations of 100 µg/mL or below. Among the tested PVPA‐*co*‐AA polymers with different VPA:AA ratios, only the co‐polymer with VPA:AA ratio no >34:66 mol %, at a concentration of no >25 µg/mL enhanced the BM‐MSCs and SaOS‐2 cells mineralization *in vitro*.

The effect of copolymer composition on the calcium chelation capacity has been investigated and related to the ability of the polymers to affect cell matrix mineralization *in vitro*. We have proposed a mechanism for the calcium chelation of PVPA‐*co*‐AA copolymers. In aqueous solution, the carboxylic and phosphonic acid groups of PVPA‐*co*‐AA will dissociate to form a negatively‐charged polyelectrolyte. These acid groups are capable of binding to divalent calcium ions. When the concentration of calcium is high, relative to the polymer concentration, inter‐polymer complexes may be formed.^25^ This occurs as a result of calcium binding to acid groups from separate polymer chains, forming electrostatic “crosslinks.” At very high calcium concentrations, an insoluble polymer‐calcium complex is formed which precipitates out of solution.^26^

There was a notable similarity of the calcium chelation capacity of each PVPA‐*co*‐AA polymer and its effect on SaOS‐2 cells mineralization *in vitro*: with the higher calcium chelation capacity, the greater effect on SaOS‐2 cells mineralization *in vitro* was achieved. It also shows the possible correlation of the calcium chelation capacity and the mineralization percentage; namely, the better mineralization effect was possibly due to the better calcium chelation capacity of the polymer. Since the process of mineralization largely utilized the surrounding calcium, this result could be due to the unique calcium chelation property of the PVPA‐*co*‐AA: At a high PVPA‐*co*‐AA concentration (50 µg/mL or higher), all free calcium in media was chelated by the PVPA‐*co*‐AA in a soluble complex, hence cells have very little calcium available to form a mineralization matrix. At a lower PVPA‐*co*‐AA concentration, total Ca^2+^ exceeds the PVPA chelation capacity; hence it formed calcium‐PVPA‐*co*‐AA precipitates on the cell surface, and this calcium rich precipitate helped the cell mineralization. This proposed mechanism could also explain the controversial effects of PVPA from other studies,^10^, ^14^, ^15^ as the different PVPA concentrations could lead to completely different effects in bone mineralization.

Different appearances of the polymer‐calcium complex at different VPA:AA ratios were found in our research. The mechanism for the formations of different polymer‐calcium complexes is not well understood, particularly in relation to polymer‐mediated mineralization.^27^ It is known that the charge, ionic composition and molecular weight can affect the size and shape for the formation of calcium phosphate crystals.^28^ However, there are limited studies regarding the thermodynamics that underpin the process.^29^ It was observed that the composition of a copolymer from AA and VPA could be optimized to improve the chelation capacity of either AA or VPA individually. 34 mol % PVPA‐*co*‐AA demonstrated the greatest calcium chelation capacity and, consequently, was associated with the formation of dense calcium‐rich, nodule‐like agglomerates.^30^ It is not clear whether the polymer itself improved cell‐mediated mineralization or whether the formation of Ca‐polymer complexes improved the distribution and density of mineralized matrix formation. However, it was apparent that there appeared to be a link between the type and concentration of polymer used in association with cell culture media, to enhance the osteogenic response of both an osteoblast‐like cell‐line and hBM‐MSCs.^31^ Previously, Chou et al. described that the bone sialoprotein (BSP) and osteocalcin (OCN) expression of MC3T3 cells (osteoblastic cell line) were affected by the size of apatite crystals. Importantly, greater expression of both BSP and OCN were shown on larger crystals.^32^, ^33^ In our study, we demonstrate that both the polymer ratio and concentration affect the degree of mineralization and consequently, an optimal polymer‐calcium complex could be achieved to promote osteogenic differentiation and mineralization.

It has been shown that the osteogenic effect of P‐34 on hBM‐MSCs and SaOS‐2 cells were both concentration dependent, and at the optimized concentration (5 µg/mL and 10 µg/mL concentration) P‐34 treatment not only significantly increased *in vitro* mineralization at day 7 and the collagen synthesis at day 14 in SaOS‐2 cells, but also significantly increased alkaline phosphatase activity, *in vitro* mineralization, and collagen synthesis at the relevant time points in hMB‐MSCs. Interestingly, our PCR results suggested that the osteogenic effects on SaOS‐2 cells and hMB‐MSCs were from different mechanisms.

The PCR result showed that no difference was found in osteogenic genes expression in SaOS‐2 cells between the P‐34 treatment and control groups; suggesting that the P‐34 does not affect SaOS‐2 (mature osteoblast cells) gene expression. In contrast, all osteogenic gene expression in the hBM‐MSCs culture were increased with the P‐34 treatment. This is an interesting finding; as the mineralization results suggested that although P‐34 increased mineralization on both SaOS‐2 cells and hBM‐MSCs at the optimized concentration, the underlying mechanisms for both cells were probably different. The osteoconductivity of P‐34, particularly the increased *in‐vitro* mineralization in SaOS‐2 cells with P‐34 treatment, was probably due to the PVPA‐*co*‐AA chemical structure, which mimics the action of bisphosphonates, due to a similar chemical structure and the ability of the PO_3_H_2_ group to chelate Ca^2+^ ions, essentially forming a “bone hook,”^11^ with the potential to enhance bone mineralization. Furthermore, bisphosphonates exert stimulatory effects on MSCs proliferation and osteogenic differentiation.^34^ As PVPA‐co‐AA has the chemical structure which resembles bisphosphonates, the polymer may mimic the action of bisphosphonate on hBM‐MSCs through as yet an unknown mechanism. More importantly, here we have shown for the first time an osteoinductive effect of PVPA‐*co*‐AA on hBM‐MSCs. Interestingly this effect was not only achieved by calcium chelation of the PVPA‐*co*‐AA chemical structure, but P‐34 also enhanced osteogenic differentiation of hBM‐MSCs at molecular level influencing osteogenic gene expression.

## CONCLUSION

This is the first study to report the direct osteogenic effect of PVPA‐*co*‐AA polymer on MSCs and osteoblasts. With the different VPA:AA ratios in the PVPA‐*co*‐AA polymer, different osteogenic effects were observed. Among the formulations investigated, the copolymer with 34% VPA (%mol) demonstrated the best osteogenic effects, which not only enhanced osteoconductivity in SaOS‐2 cells (mature osteoblast‐like cells), but also promoted osteoinductivity in hBM‐MSCs. We also found a strong correlation between the osteogenic effects and the calcium chelation capacity of the investigated co‐polymers. More importantly, this is the first study to report that different concentrations of PVPA‐*co*‐AA could also lead to differential effects on bone mineralization, together with a possible mechanism. This proposed mechanism can also explain the conflicting effects of PVPA from other studies. Moreover, with the optimized P‐34 concentration defined in this study, further work is underway to investigate the performance of P‐34 incorporated tissue engineering scaffold *in vitro* and *in vivo*. The knowledge will be critical when incorporating PVPA‐co‐AA polymers in the design of novel bioactive polymeric tissue engineering scaffolds for future clinical applications.

## NOTE

The authors declare no competing financial interest.
